# FBA reveals guanylate kinase as a potential target for antiviral therapies against SARS-CoV-2

**DOI:** 10.1093/bioinformatics/btaa813

**Published:** 2020-12-29

**Authors:** Alina Renz, Lina Widerspick, Andreas Dräger

**Affiliations:** 1 Computational Systems Biology of Infections and Antimicrobial-Resistant Pathogens, Institute for Bioinformatics and Medical Informatics (IBMI); 2 Department of Computer Science, University of Tübingen, Tübingen 72076, Germany; 3 German Center for Infection Research (DZIF), partner site Tübingen, Germany

## Abstract

**Motivation:**

The novel coronavirus (SARS-CoV-2) currently spreads worldwide, causing the disease COVID-19. The number of infections increases daily, without any approved antiviral therapy. The recently released viral nucleotide sequence enables the identification of therapeutic targets, e.g. by analyzing integrated human-virus metabolic models. Investigations of changed metabolic processes after virus infections and the effect of knock-outs on the host and the virus can reveal new potential targets.

**Results:**

We generated an integrated host–virus genome-scale metabolic model of human alveolar macrophages and SARS-CoV-2. Analyses of stoichiometric and metabolic changes between uninfected and infected host cells using flux balance analysis (FBA) highlighted the different requirements of host and virus. Consequently, alterations in the metabolism can have different effects on host and virus, leading to potential antiviral targets. One of these potential targets is guanylate kinase (GK1). In FBA analyses, the knock-out of the GK1 decreased the growth of the virus to zero, while not affecting the host. As GK1 inhibitors are described in the literature, its potential therapeutic effect for SARS-CoV-2 infections needs to be verified in *in-vitro* experiments.

**Availability and implementation:**

The computational model is accessible at https://identifiers.org/biomodels.db/MODEL2003020001.

## 1 Introduction

In December 2019, an outbreak of pneumonia in Wuhan, Hubei province, in China, has aroused the interest of the international community by showing alarming similarities to the outbreaks caused by other β-*coronaviruses* (β-CoV) like the Severe Acute Respiratory Syndrome (SARS) virus (Huang *et al.*, 2020; Hui *et al.*, 2020).

The febrile respiratory illness caused by the novel coronavirus (SARS-CoV-2) is thought to have spread as a zoonosis from the Huanan Seafood Wholesale Market, which was as a consequence shut down on January 1, 2020 to prevent further transmission events (Hui *et al.*, 2020).

On January 7, first isolation and subsequent deep-sequencing of SARS-CoV-2 from the human lower respiratory tract samples have made the genetic sequence of the virus available to the public by January 12, 2020, thus allowing for the identification of the virus as a Group 2B β-CoV (Huang *et al.*, 2020; Hui *et al.*, 2020). SARS-CoV-2 has 82% sequence similarity with the SARS virus, which has caused an outbreak originating in China in 2002 (Hui *et al.*, 2020; Zhang *et al.*, 2020). The outbreak in 2002 has peaked at a total of 8098 documented cases with a case fatality rate of 9.6% (Huang *et al.*, 2020; Hui *et al.*, 2020). In contrast, the ongoing SARS-CoV-2 has reached, at time of writing, a total of ∼3 million cases worldwide and caused over 200 000 deaths (John Hopkins University, 2020; WHO, 2020). The resemblance and the severe global health threat have initiated a swift and determined implementation of public health measures by the Chinese Authorities (Chen *et al.*, 2020; Hui *et al.*, 2020).

While human-to-human transmissions in a nosocomial setting were the primary route of transmission of the SARS virus outbreak, Chinese horseshoe bats have been identified as putative primary reservoir and source of zoonosis for SARS (Huang *et al.*, 2020; Hui *et al.*, 2020; Paraskevis *et al.*, 2020). Moreover, Himalayan palm civets, raccoon dogs and Chinese ferret badgers from the Guangdong wet markets (live animal retail markets) were identified as intermediate reservoirs of zoonosis (Hui *et al.*, 2020; Perlman and Netland, 2009). In the case of SARS, the switch to the human host was allowed by an adaption of the receptor binding domain of the spike (S) protein, conferring to binding capabilities to the human angiotensin-converting enzyme 2 (ACE2) (Huang *et al.*, 2020; Perlman and Netland, 2009). For the SARS-CoV-2, there was initially no proof of efficient human-to-human transmission, however, the rapid increase in cases and distinct clustering of the disease have made it clear that an efficient transmission route in-between humans exists (Chan *et al.*, 2020; Hui *et al.*, 2020). Moreover, Paraskevis *et al.* (2020) have suggested, based on full-genome evolutionary analysis, a zoonosis of SARS-CoV-2 from bats to humans. Accordingly, Letko *et al.* have recently identified ACE2 as the SARS-CoV-2 entry receptor (Letko and Munster, 2020).

As mentioned above, the SARS-CoV-2 belongs to the subfamily of β-CoV (Huang *et al.*, 2020; Hui *et al.*, 2020). The members of the family are enveloped, single-stranded RNA (ssRNA) viruses with a positive polarity genome of up to 34 kb (Chen *et al.*, 2020; Huang *et al.*, 2020; Zhang *et al.*, 2020). Replication of the RNA genome is performed via an RNA-dependent RNA polymerase (RdRP) in double-membrane vesicles (DMVs), modified to form a reticulovesicular network (Chen *et al.*, 2020; de Wilde *et al.*, 2015; Perlman and Netland, 2009). In the DMVs, the 16 non-structural proteins (NSPs) are directly expressed as polyproteins pp1a and pp1ab from the (+)RNA genome (Chen *et al.*, 2020). Processing of pp1a/1ab by the viral main protease (M^pro^) is essential to form the replication-transcription complex for the subsequent expression of the viral structural proteins (Chen *et al.*, 2020; Perlman and Netland, 2009).

In total, the NSPs constitute two thirds of the genome’s coding capacity (Chen *et al.*, 2020). The rest encodes for structural proteins, such as the spike (S), membrane (M) and envelope (E) proteins, which cover a helical nucleocapsid made up of the nucleocapsid (N) proteins (Chen *et al.*, 2020; Perlman and Netland, 2009). Unlike NSPs, the structural and additional accessory proteins of *coronaviruses* require synthesis as subgenomic messenger RNAs via discontinuous transcription from (-)RNA templates (Chen *et al.*, 2020).

Clinically, the SARS-CoV-2 seems to be milder than the former SARS virus, although they share the symptoms of febrile illness and pneumonia (Chen *et al.*, 2020; Huang *et al.*, 2020; Hui *et al.*, 2020). It was recently shown that the SARS-CoV-2 does not only infect lower respiratory tract cells, but also upper cells in the pharyngeal region (Chen *et al.*, 2020; Huang *et al.*, 2020). Moreover, many *coronaviruses* also infect macrophages and subsequently inhibit an interferon-stimulated genes-mediated antiviral response, increasing immune evasion and pathogenicity (Chen *et al.*, 2020; Cheung *et al.*, 2005; Deng *et al.*, 2017; Huang *et al.*, 2020).

By now, no antiviral treatment for *coronaviruses* has been proven efficacious in a clinical trial (Chen *et al.*, 2020; Huang *et al.*, 2020). Recently, Zhang *et al.* (2020) have found α-ketoamides to be broad-spectrum inhibitors of *coronaviruses* by tissue-dependently inhibiting the M^pro^ of SARS in Vero cells. This inhibition has occurred in a micromolar EC_50_ range, indicating α-ketoamides to be a potential antiviral for the SARS-CoV-2 (Zhang *et al.*, 2020). However, since no therapy or vaccination is available for clinical use, the current standard of care for a SARS-CoV-2 infection is limited to the supportive treatment of symptoms (Chen *et al.*, 2020; Huang *et al.*, 2020).

As no antiviral treatment is currently available for *coronaviruses*, the identification of potential antiviral targets is of great interest. One possibility of identifying new targets is the analysis of metabolic changes in infected cells. Aller *et al.* introduced a procedure for integrated human-virus metabolic models to predict host-based antiviral targets against Chikungunya, Dengue and Zika viruses. The analysis of the integrated human-virus models predicted inhibiting effects of constrained host-reactions on virus production. These predictions included already known targets of existing antiviral drugs, demonstrating the applicability of such analysis methods (Aller *et al.*, 2018).

In our study, we integrated and analyzed a human genome-scale metabolic model (GEM) infected with SARS-CoV-2. As it is shown that *coronaviruses* infect alveolar macrophages (Cheung *et al.*, 2005; Deng *et al.*, 2017; Joel Funk *et al.*, 2012), and Bordbar *et al.* (2010) already published an extensive metabolic model of human alveolar macrophages, this model was used as a host model. For SARS-CoV-2, a virus biomass objective function (VBOF) was generated according to Aller *et al.* (2018). Subsequent analysis of the integrated host-virus model revealed potential targets for antiviral therapies.

## 2 Materials and methods

The methods used in this article are based on the article by Aller *et al.* (2018). Methods and analyses were adapted and expanded for the coronavirus SARS-CoV-2.

### 2.1 Generation of VBOF

Analogous to the biomass production or maintenance function in prokaryotic or eukaryotic metabolic models, the VBOF is a pseudo-reaction simulating the production of virus particles. It comprises nucleotides, amino acids and associated energy metabolites required for the production of the virus particles. Due to a lack of knowledge, the stoichiometric information of the virus envelope, and the dynamic information of lipids are not included in the VBOF. Hence, virus entry or exit cannot be modeled. The generation of the VBOF was performed following the seven steps described by Aller *et al.* (2018).

1. *Step 1: Virus genome and protein information*. The recently published virus genome and protein sequence were obtained from the NCBI database (Geer *et al.*, 2010) with the accession number NC_045512.2 in February 2020. Essential for the calculation of the nucleotide count is the classification of the SARS-CoV-2 in the Baltimore Classification System (Baltimore, 1971) that characterizes viruses based on the replication method. *Coronaviruses* fall into the Group IV classification. These viruses replicate their positive single-stranded RNA (+ssRNA) genomes via a complementary negative ssRNA. The nucleotide counts of the positive strand can be taken from the genome sequence. The nucleotides of the negative strand can be calculated by counting the complementary nucleotides of the positive strand. Both nucleotides are summed up to receive the total nucleotide count.SARS-CoV-2 has a total of 12 proteins, four structural proteins, and 8 NSPs. Structural and NSPs are not expressed equally, and this ratio needs to be considered during the calculation of the VBOF. To this date, no information about the copy number of structural proteins in *Coronaviruses* is reported.
2. *Step 2: Nucleotide investment*. The nucleotide count of the virus genome and its replication intermediate are summed per nucleotide. The genome copy number (*Cg*) was assumed to be one. According to Aller *et al.*, the moles of nucleotides were converted into grams of nucleotides per mole of the virus. After similar calculations of the amino acids and the calculation of the total molar weight of the virus based on nucleotide and amino acid content, the stoichiometric coefficients of each nucleotide in the VBOF were calculated.
3. *Step 3: Amino acid investment*. Analogous to the nucleotide investment, the stoichiometric coefficient of each amino acid was calculated. Instead of the genome copy number, a copy number for structural and NSPs is required. As already mentioned, the copy number of structural proteins is not reported to date. Therefore, we repeated all analyses described in this article for copy numbers of structural proteins *C*_sp_=200, 500, 800 and 1200. With the information of the total count of each amino acid, weighted by the copy number of structural proteins, and the inclusion of the respective molar mass, the total viral molar mass was calculated (see also Step 6) to eventually calculate the stoichiometric coefficient for every amino acid.
4. *Steps 4 and 5: Adenosine triphosphate (ATP) requirements, and pyrophosphate liberation*. The calculations of the ATP requirements for the polymerization of amino acids and the calculation of pyrophosphate (PPi) liberation from the nucleotide polymerization were performed as described by Aller *et al.* based on the results of the previous steps. The constants $k_{\text{ATP}}=4$ and $k_{\text{PPi}}=1$ were chosen, as suggested by Aller *et al.* $k_{\text{ATP}}$ is the required amount of four ATP molecules for the polymerization of amino acid monomers. The constant $k_{\text{PPi}}$ stands for the liberation of one diphosphate molecule per polymerization of nucleotide monomers.
5. *Steps 6 and 7: Total viral molar mass and final construction of the VBOF*. The total molar mass of SARS-CoV-2 and the final VBOF were calculated and constructed in accordance with Aller *et al.*

### 2.2 Integration of the SARS-CoV-2 virus into *i*AB-AMØ-1410

Since Coronaviridae infect alveolar macrophages (Cheung *et al.*, 2005; Deng *et al.*, 2017), the cell-specific GEM of human alveolar macrophages *i*AB-AMØ-1410 was utilized. This GEM was published in 2010 by Bordbar *et al.* (2010) to give insight into human alveolar macrophages infected with *Mycobacterium tuberculosis*. The cell-specific GEM was constructed based on the human metabolic reconstruction Recon 1 (Duarte *et al.*, 2007). It consists of 3394 reactions and 2572 metabolites. The model was downloaded in SBML Level 2 Version 4 format (Hucka *et al.*, 2008) and upgraded to SBML Level 3 Version 1 format (Hucka *et al.*, 2018) using libSBML (Bornstein *et al.*, 2008). The VBOF was incorporated into the existing macrophage model, using flux bounds of 0 and 1000 as lower and upper bounds. Additionally, aggregated subsystems were added to the reactions as additional information for further analyses (Aller *et al.*, 2018; Zielinski *et al.*, 2015).

### 2.3 Identification of stoichiometric differences

The human alveolar macrophage model *i*AB-AMØ-1410 included now two pseudo-reactions for the production or maintenance of the virus and hosts biomass, respectively. The stoichiometric coefficients of shared metabolites within these pseudo-reactions can be compared to identify differences in nucleotide or amino acid requirements. First, the individual amino acid and nucleotide stoichiometric coefficients were normalized against the sum of all metabolites present in the respective biomass objective function (BOF), except for the metabolites for energy requirements. Then, the fold-change (FC) of the normalized amino acid or nucleotide was calculated:

(1) $${\text{FC}}_{i}={\text{log}}_{2}\left(\frac{S_{i}^{V}/\sum_{k}S_{k}^{V}}{S_{i}^{H}/\sum_{k}S_{k}^{H}}\right)\;,$$

with index *i* over nucleotides or amino acids and *k* over all biomass precursors (except for energy requirements). Subscripts *H* and *V* represent the use of either the host or virus biomass function. Positive values imply higher usage of nucleotides or amino acids in the virus compared to the host, while negative values imply a lower usage.

### 2.4 Flux balance analysis and flux variability analysis for the comparison of host- and virus-optimized states

The integration of the VBOF into the *i*AB-AMØ-1410 model paved the way for the analysis of metabolic changes between host and virus-optimized states. To identify an optimal state, an objective function that is optimized needs to be defined. In general, every reaction in the GEM can serve as objective function. However, biologically meaningful objective functions depend on the research question. For analyses of the growth or survival of the studied organism or cell, biomass production or maintenance reactions can be introduced into the model as pseudo-reactions. In industrial settings, the production of a specific metabolite might be of interest, and hence its production reaction can be set as the objective function. The defined objective function is then optimized with flux balance analysis (FBA). FBA is a mathematical approach using linear optimization to analyze the flow of metabolites through a metabolic network while optimizing for an objective function (Orth *et al.*, 2010). This objective function is optimized under a set of constraints. These constraints are, on the one hand, defined by the stoichiometry of the reactions, and, on the other hand, by limitations of reaction fluxes through upper and lower bounds. Hence, not only the biomass production or maintenance reaction can be adapted to a specific organism or cell type, but also the environment, in which it occurs, can be adapted accordingly, e.g. by constraining exchange fluxes. In this work, we optimized the host–virus integrated *i*AB-AMØ-1410 model for either the host biomass maintenance function or the VBOF using FBA.

Since the solutions calculated by FBA are often not unique, the flux variability analysis (FVA) is a method to identify alternate optimal solutions. In FVA, the maximum and minimum possible flux for each reaction in the network is evaluated while constraining the objective value equal or close to the optimal flux (Orth *et al.*, 2010). As for the FBA, the FVA was conducted for both the host- and virus-optimized states. The results of the FVA were used in the subsequent host-derived enforcement to define the upper and lower bounds.

#### 2.4.1 Copy number analysis

As the copy number of structural proteins in SARS-CoV-2 or *coronaviruses*, in general, is not reported so far, we evaluated the effect of different *C*_sp_ values on the growth rate in the virus-optimized state. To do so, we varied the copy number *C*_sp_ between 1 and 1500. The variation of *C*_sp_ leads to changing stoichiometric coefficients in the VBOF. Since the VBOF is optimized in the virus-optimized state, changing VBOF reactions can lead to changing optimization results.

#### 2.4.2 Optimization analysis

The host-virus integrated model holds both the host biomass maintenance and the VBOF reaction. First, each biomass reaction was optimized individually using FBA. The objective function is defined by setting the objective coefficient to 1. In general, only one reaction (the objective function) has an objective coefficient of 1. However, one can also set the objective coefficient of several reactions to 1 to optimize for both reactions. Hence, the objective coefficient of both biomass functions from host and virus was set to 1 to optimize for both functions. Last, we used COBRApy’s (Ebrahim *et al.*, 2013) tailored objectives option to weight the influence of the two biomass reactions on the objective function. To do so, we created a linear combination of the two biomass reactions weighted by factors varying between 0 and 1, and not exceeding 1 in total. When the host maintenance function weighted 0.2, the VBOF had a weight of 0.8 in the objective function. For each combination, the model was optimized using FBA, and the flux through the two biomass reactions was reported. This analysis was repeated using the results from the host-derived enforcement analysis by adapting the bounds of the reported reactions (Section 2.5.2).

#### 2.4.3 Metabolic analysis

The metabolic analysis was performed for the host- and virus-optimized states. The fluxes of each reaction in the two optimized states were compared by calculating the FC_*r*_:

(2) $$FC_{r}={\text{log}}_{2}\left(F_{r}^{V}/F_{r}^{H}\right)\;,$$

where the indexation *r* is over all reactions of the model, and the superscript *H* indicates flux values from the host and *V* from the virus-optimized models. The FCs were aggregated into the included subsystems.

### 2.5 Antiviral target identification

For the identification of potential antiviral targets that preferentially alter the virus growth rate while maintaining the host’s biomass maintenance, each reaction in the model was evaluated using two different approaches.

#### 2.5.1 Reaction knock-out

The first approach is a reaction knock-out that is already implemented in COBRApy. The single_reaction_deletion function subsequently sets both bounds of every reaction to zero and optimizes for the chosen objective function. We ran this function twice, once with the host maintenance reaction as the objective function and once with the VBOF. Possible targets were only reported when the growth of the virus was diminished (<99% of its initial growth rate) and when the growth rate of the virus was below the growth rate of the host-optimized state.

#### 2.5.2 Host-derived enforcement

In the second approach, the effects of changes in flux ranges of the reactions on the VBOF are analyzed, while the metabolic system of the host-optimized state is maintained. To change the flux bounds ensuring maximal biomass maintenance of the host while optimizing for the VBOF, the results of the FVA were utilized. For each reaction, the flux range was defined as described by Aller *et al.* The corresponding minimum (*F*^–)^ and maximum (*F*^+^) flux values from the FVA of the host (*H*) and virus (*V*) optimization and their relation define the minimum ($\varepsilon^{-}$) and maximum ($\varepsilon^{+}$) flux values. Since the cases suggested by Aller *et al.* did not cover all reactions, we added the two more conditional arguments cases 3 and 7:

$$\{\begin{array}{cc} \;\;\text{continue} & \text{if}\;F_{H}^{+}=F_{V}^{+}\wedge F_{H}^{-}=F_{V}^{-}\;\;\;\;\;\;\;\;\;\;\;\;\;\;\;\;\;\;\;\;\;\;\;\;\;\;\;\;\;(3)\\ \begin{array}{c} \varepsilon^{+}=F_{H}^{+}\\ \varepsilon^{-}=F_{H}^{+}-\frac{F_{H}^{+}-F_{V}^{+}}{2} \end{array} & \text{if}\;F_{H}^{+}>F_{V}^{+}\wedge F_{H}^{-}\geq F_{V}^{-}\;\;\;\;\;\;\;\;\;\;\;\;\;\;\;\;\;\;\;\;\;\;\;\;\;\;\;\;(4)\\ \begin{array}{c} \varepsilon^{+}=F_{H}^{-}-\frac{F_{H}^{-}-F_{V}^{-}}{2}\\ \varepsilon^{-}=F_{H}^{-} \end{array} & \text{if}\;F_{H}^{+}\leq F_{V}^{+}\wedge F_{H}^{-}<F_{V}^{-}\;\;\;\;\;\;\;\;\;\;\;\;\;\;\;\;\;\;\;\;\;\;\;\;(5)\\ \begin{array}{c} \varepsilon^{+}=F_{H}^{+}\\ \varepsilon^{-}=F_{H}^{-} \end{array} & \text{if}\;F_{H}^{+}<F_{V}^{+}\wedge F_{H}^{-}>F_{V}^{-}\;\;\;\;\;\;\;\;\;\;\;\;\;\;\;\;\;\;\;\;\;\;\;\;\;\;(6)\\ \begin{array}{c} \varepsilon^{+}=F_{H}^{+}-\frac{F_{H}^{+}-F_{V}^{+}}{2}\\ \varepsilon^{-}=F_{H}^{-}-\frac{F_{H}^{-}-F_{V}^{-}}{2} \end{array} & \text{if}\;F_{H}^{+}\leq F_{V}^{+}\wedge F_{H}^{-}\geq F_{V}^{-}\;\;\;\;\;\;\;\;\;\;\;\;\;\;\;\;\;\;\;\;\;\;\;\;\;(7) \end{array}$$

The flux values $\varepsilon^{-}$ and $\varepsilon^{+}$ are set as upper and lower bounds of the corresponding reactions and the model was optimized for the VBOF. The resulting optimization result was compared to the original VBOF growth rate. When the growth rate with adapted bounds was below the threshold of 80% of the initial growth rate, the reaction was reported as potential antiviral target.

## 3 Results

We developed a GEM of a human macrophage infected with the SARS-CoV-2. To integrate the virus into the human macrophage, a virus biomass reaction representing the virus particle production was added to the model. This virus biomass reaction was generated based on the nucleotide and amino acid sequence.

### 3.1 Stoichiometric differences

**Fig. 1.**
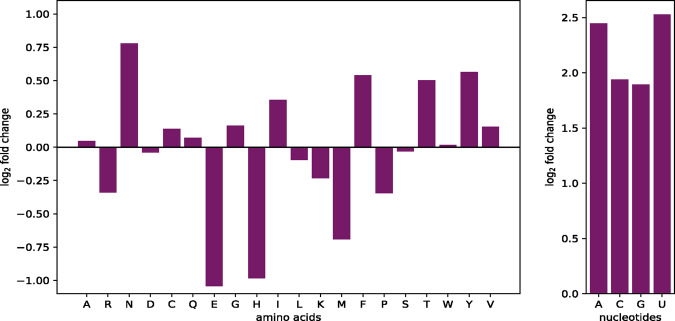
FC differences in amino acid and nucleotides usage. The stoichiometric coefficients of the alveolar macrophage biomass maintenance function and the VBOF are compared using Equation (1). The left panel displays the FC of all 20 proteinogenic amino acids. The one letter code of the amino acids is used for labeling the *x*-axis. The right panel displays the FC of the four RNA nucleotides. The one letter code of the RNA nucleotides is used for labeling the *x*-axis

The human alveolar macrophage biomass maintenance function is comprised of several macromolecules, including amino acids, deoxyribonucleic acid (DNA) and RNA nucleotides, components for the energy requirements and others, such as fatty acids or phospholipids. In contrast, the VBOF only consists of amino acids, RNA nucleotides and the components for the energy requirements. The stoichiometric coefficients of amino acids and nucleotides are compared by calculating the FC. The result is visualized in Figure 1. The stoichiometric coefficients of L-asparagine (N), L-phenylalanine (F), L-threonine (T) and L-tyrosine (Y) are increased. In contrast, the coefficients of L-glutamate (E), L-histidine (H) and L-methionine (M) are reduced. These findings might indicate an up- or down-regulation of the respective metabolic pathways.

### 3.2 Influence of the copy number of the structural proteins

**Fig. 2.**
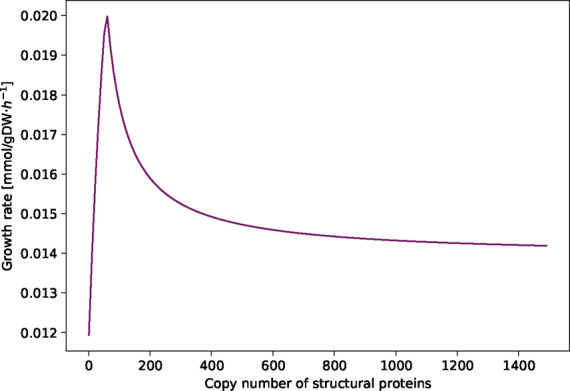
Influence of $C_{sp}$ on the growth rate. The copy number of structural proteins *C*_sp_ in *coronaviruses* is not yet reported in literature. Hence, its influence on the growth rate in the virus-optimized state is evaluated by varying *C*_sp_ between 1 and 1500. For each copy number, the integrated host-virus model was optimized for the VBOF. For *C*_sp_ values between 58 and 1500, the growth rate decreases

The calculations for obtaining a virus VBOF include the parameter for the copy number of the structural proteins (*C*_sp_). The copy number of structural proteins for some viruses, such as *Alpha*- and *Flaviviruses*, is known and ranges from 180 for *Flaviviruses* (Mukhopadhyay *et al.*, 2005) to 240 for *Alphaviruses* (Strauss and Strauss, 1994). *Coronaviruses* have four major structural proteins: the envelope (E) protein, membrane (M) protein, nucleocapsid (N) protein and the spike (S) protein (Masters, 2006; Mortola and Roy, 2004; Wang *et al.*, 2017). However, the copy number of those structural proteins is not reported so far. To evaluate the influence of the copy number on the modeling results, we varied the parameter *C*_sp_ between 1 and 1500. As shown in Figure 2, the optimization result of the VBOF depends on the copy number. The growth rate increases for copy numbers ranging from 1 to 58 and decreases for higher copy numbers. For very high copy numbers of structural proteins, the growth rate seems to reach a steady state.

*Coronaviruses* have a diameter of ∼80–120 nm (Guy *et al.*, 2000), while *Alpha*- and *Flaviviruses* have only a diameter of ∼40–80 nm and 30–50 nm, respectively (Fraenkel-Conrat and Wagner, 1974). However, not only the virus size is larger, but also the size of its structural proteins. Since no value for the structure protein copy number in *coronaviruses* was available, we continued our analyses with a *C*_sp_ value of 500 and verified the results with *C*_sp_ values of 200, 800 and 1200.

### 3.3 Optimization of host and virus metabolism

**Fig. 3.**
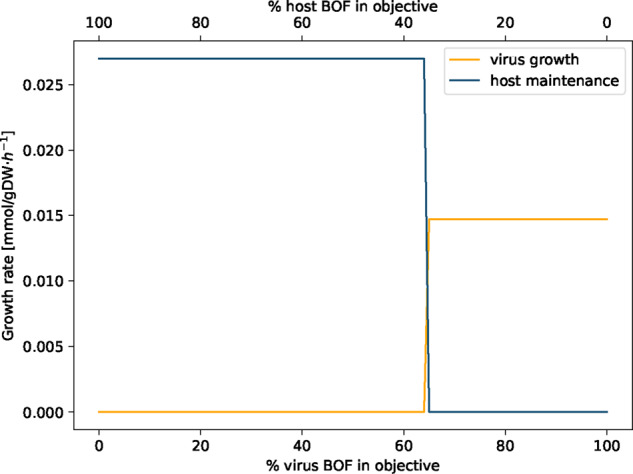
Linear combination of weighted biomass functions. The objective function was defined as linear combination of the host maintenance and VBOF by weighting the proportion of the respective biomass functions between 0 and 1. The sum of the two weights always sums up to 1. At 65% of virus BOF contribution to the objective expression, a switch between host maintenance and virus production occurs

Potential changes in the human alveolar macrophage metabolism during virus infection were first examined by analyzing the following two scenarios: (i) the host cell is not infected and the metabolic system is optimized for the already included maintenance biomass reaction (host optimization). This scenario reflects the normal physiological state of human alveolar macrophages; (ii) the host cell is infected by the virus and is optimized solely for the production of the virus particles (virus optimization). The host optimization results in a biomass maintenance flux of 0.0267 mmol/(gDW⋅h), while the virus optimization returns a flux of 0.0147 mmol/(gDW⋅h). When defining both the host maintenance and the virus biomass function as objective functions by assigning both an objective coefficient of one simultaneously, the model only optimizes for the host maintenance reaction while the flux through the VBOF is zero. Last, the optimization result of host and virus metabolism was compared using a new constraint. With varying percentages of the host maintenance reaction and the VBOF on the objective expression, the effect on the respective biomass maintenance or growth function was investigated. The model does not predict an equilibrium state, where both the host maintenance reaction and the VBOF are active. As displayed in Figure 3, the switch between the maintenance of the host metabolism and the growth of the virus is at 65% of VBOF contribution to the objective expression. This switch in biomass production is rather insensitive to the structural proteins’ copy number. For *C*_sp_=200, the switch occurs at 63% virus BOF contribution, and for *C*_sp_=800 and *C*_sp_=1200 at 66%.

### 3.4 Metabolic changes in alveolar macrophages after virus infection

**Fig. 4.**
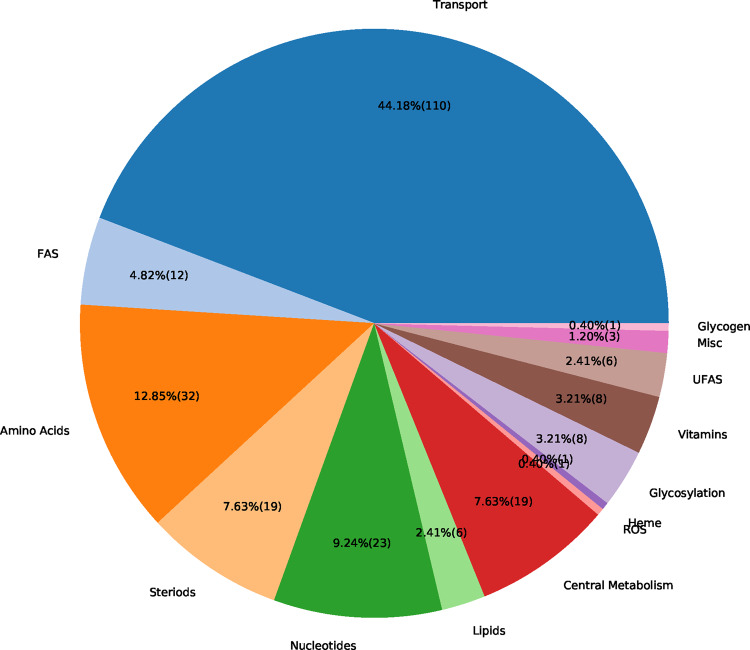
Altered reactions in virus-optimized state sorted by subsystems. The integrated virus–host model was optimized for the host maintenance and VBOF using FBA. The FC was calculated using Equation (2) and the reactions with FCs were grouped into the aggregated subsystems. Many altered reactions belong to the transport subsystem, or amino acid and nucleotide metabolism

The flux distribution for the host- and the virus-optimized states were compared using FBA and FC calculations. For 256 reactions (7.8% of all model reactions), a FC was calculated. As expected from the stoichiometric analysis, reactions related to amino acid and nucleotide metabolism were altered. The main portion of changed reactions consists of transport reactions. However, also several reactions from other subsystems, such as the steroid, fatty acid synthesis and central metabolism, are altered. An overview of altered reactions concerning their FC is given in Figure 4. One needs to keep in mind that the FC can only be calculated if neither of the reaction fluxes in the host- and virus-optimized state was zero. Hence, we calculated the absolute change for reactions that have either in the host- or the virus-optimized state a flux of zero. A total of 97 reactions (2.9% of all model reactions) either turned on a previously turned off reaction or vice versa. For example, in the virus-optimized state, the virus turned off 14 reactions related to lipid metabolism.

### 3.5 Identification of metabolic targets for antiviral strategies

FBA and FVA can be used to identify metabolic targets for antiviral strategies by comparing the host- and virus-optimized states after alterations in metabolic pathways.

#### 3.5.1 Knock-out of reactions reveals first promising metabolic target for antiviral strategies

The results from the stoichiometric differences and metabolic changes in alveolar macrophages after virus infections with coronavirus SARS-CoV-2 indicate alternative host- and viral-optimal states. The diverging flux distribution in the two states provides an opportunity for the identification of potential antiviral targets. To identify potential antiviral targets that limit virus production, we implemented two different analysis methods: (i) reaction knock-outs and (ii) host-derived enforcement. The reaction knock-out revealed exactly one reaction (over all tested copy numbers of structural proteins), whose knock-out reduces the flux of the virus biomass to zero, while maintaining the host biomass maintenance at 100%: The guanylate kinase (GK1) reaction that converts ATP and guanosine monophosphate (GMP) to adenosine diphosphate and guanosine diphosphate (GDP):

(8) $$\text{ATP}+\text{GMP}\overset{\mathrm{GK}1}{⇌}\mathrm{ADP}+\mathrm{GDP}\;.$$

#### 3.5.2 Host-derived enforcement substantiates the metabolic target and reveals further points of action

This reaction is also observed as a potential target in the second analysis method, the host-derived enforcement. In this approach, the reaction fluxes are constrained to ranges derived from FVA. With flux ranges outside of the optimal state of the virus, the virus production is perturbed while the host maintenance is not affected. For structural protein copy numbers between 500 and 1200, we identified four possible targets, including the GK1 that reduced the virus growth flux to below a threshold of 80% of its initial value. Further potential targets concern the availability of the amino acids L-isoleucine (I), and L-lysine (K), either via the alteration of exchange reactions, or, in case of L-isoleucine, also via the L-isoleucine transporter. In contrast to GK1, where the flux through the reaction is down-regulated (or in case of the reaction knock-outs completely knocked out), the alterations of the mentioned exchange, and transport reactions go into the other direction: the uptake of the amino acids is enabled or even enforced, leading to the host’s maintenance while decreasing the growth of the virus to 50% of its initial growth. The enforcement of exchange reactions can be reached by a sufficient supply of the respective amino acid.

**Fig. 5.**
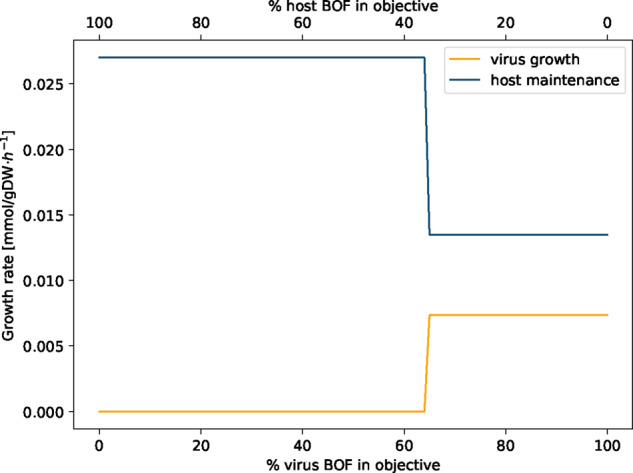
Linear combination of weighted biomass function with adapted boundaries for L-lysine exchange. The objective function was defined as linear combination of the host maintenance and VBOF by weighting the proportion of the respective biomass functions between 0 and 1. The sum of the two weights always sums up to 1. The upper and lower bounds of the L-lysine exchange were adapted based on the host-derived enforcement results (see Section 2.5.2). Even when the objective function is solely defined by the VBOF (% of virus BOF in objective =100%), the growth rate of the host still exceeds the growth rate of the virus

If the host has enough L-lysine in its environment, the maintenance of the host cell is ensured, while the growth of the virus is diminished. We repeated the analysis that evaluates the effect on the growth rates based on the percental involvement of the two biomass functions within the setting of L-lysine excess. Figure 5 illustrates the virus inhibiting effect of L-lysine availability in the environment. Even when the objective function is solely defined by the VBOF (% of virus BOF in objective =100%), the growth rate of the host still exceeds the growth rate of the virus.

Analogous to L-lysine, the availability of L-isoleucine and L-tyrosine has a positive effect on the growth of the host while decreasing the growth rate of the virus by 50%. The last potential metabolic target for antiviral strategies using the host-derived enforcement approach concerns one transport reaction of L-isoleucine (ILEtec). Enforcing a minimal import of L-isoleucine via this transport has the same effect as the other three targets and results in a diminution of 50% of the virus growth. All targets obtained from the host-derived enforcement for the structural protein copy numbers *C*_sp_=500, 800 and 1200 are summarized in Table 1.

**Table 1.** Host-derived enforcement of reactions reduced the growth of the virus

Reaction ID	Growth host (mmol/gDW⋅h^-1^)	Growth virus(mmol/gDW⋅h^-1^)	Regulation
GK1	0.027	0.00736	Down
EX_ile__L(e)	0.027	0.00736	Up
EX_lys__L(e)	0.027	0.00736	Up
ILEtec	0.027	0.00736	Up

*Note*: The host-derived enforcement analysis (see Section 2.5.2) disclosed reactions reducing the virus growth (initial growth rate 0.0147 mmol/gDW⋅h^-1^), while maintaining the host’s growth rate at 100%.

The analysis of the host-derived enforcement for *C*_sp_=200 showed among Table 1 listed 29 reactions as potential antiviral targets that decrease the virus growth below the threshold of 80%. While the four already known targets again reduced the growth rate by 50%, the growth reduction of the other targets varies between 70% and 80% of the initial value. Of the 25 new targets, almost half (12 reactions) are associated with nucleotide metabolism. Only four further reactions are affecting the amino acid metabolism and transport mechanisms, respectively. The other reactions affected are part of the central metabolism and miscellaneous reactions.

### 3.6 Existing drugs can target the predicted reactions

For the identified potential targets for antiviral therapies, we have searched for existing drugs or compounds.

#### 3.6.1 Direct inhibition of GK1

As GK1, also known as guanosine monophosphate kinase, was found to be an essential factor for viral growth in this study, the inhibition of the enzyme may be a feasible target in SARS-CoV-2 therapy. The enzyme catalyzes the reversible turnover of GMP or deoxyguanosine monophosphate to GDP or deoxyguanosine diphosphate by binding and transferring a phosphoryl from ATP to GMP (Hible *et al.*, 2006; Khan *et al.*, 2019). GK1 has a highly conserved structure, with a core domain, a GMP-binding domain, a lid domain with catalytic residues, as well as an ATP binding (P)-loop (Hible *et al.*, 2006; Khan *et al.*, 2019). Through activation of ganciclovir in herpes virus treatment and 6-thioguanine or 6-mercaptopurine activation in tumor treatment, GK1 plays an essential role in diverse treatment strategies (Hible *et al.*, 2006; Khan *et al.*, 2019).

Based on the results of this study, we propose a direct inhibitor of GK1 to harbor the potential of SARS-CoV-2 inhibition. Interestingly, such inhibitors have already been described in the context of antiviral therapies (Hible *et al.*, 2006; Jain *et al.*, 2016; Khan *et al.*, 2019; Navé *et al.*, 1992). For instance, Hible *et al.* have demonstrated P1-(5’-adenosyl) P5-(5’-guanosyl) pentaphosphate (Ap5G) to be a potent, bi-substrate inhibitor of GK1 (Hible *et al.*, 2006; Jain *et al.*, 2016). Although Hible *et al.* have only shown the high potency of the inhibition in *Escherichia coli*, Khan *et al.* have demonstrated the binding of Ap5G to introduce a complete closure of the human GK1, indicating inaccessibility of the substrates upon inhibitor binding (Hible *et al.*, 2006; Jain *et al.*, 2016; Khan *et al.*, 2019).

Potent GK1 inhibitors have also been presented by Navé *et al.* (1992). Accordingly, 9-phosphonoalkyl derivates, such as 9-(6-phosphonohexyl)guanine and 9-(6,6-difluoro-6-phosphonohexyl)guanine, impede GK1 activity via competitive inhibition of GMP and non-competitive inhibition of ATP (Navé *et al.*, 1992).

#### 3.6.2 Acyclonucleotide analogues require GK1 activation

In subsequent work, Navé *et al.* (1995) have tested the antiviral activity of 9-phosphonopentenyl derivatives of guanine operating as acyclonucleotide analogues. Acyclonucleotide analogs are pro-drugs, which require the activation by GK1 to form nucleoside triphosphate analogues (Navé *et al.*, 1995). These are known to inhibit viral DNA polymerases via chain termination in diverse herpes virus and retrovirus infections (Navé *et al.*, 1995). In accordance with this, the authors have identified vinyl phosphonates (E)-9-(5-Phosphonopent-4-enyl)guanine and (E)-9-[3-(hydroxymethyl)-5-phosphonopent-4-enyl]guanine to be inhibitors of the human immunodeficiency virus 1 (HIV-1) and human cytomegalovirus (Navé *et al.*, 1995).

As this study has found increased flux through GK1 in SARS-CoV-2 infected cells, a process of pro-drug activation relying on GK1 may allow for increased activation of terminating nucleoside triphosphate analogs in infected cells compared to healthy cells. However, *coronaviruses* are +ssRNA viruses replicated by an RdRP, unlike most viruses targeted by available acyclonucleotide analogs, such as ganciclovir and acyclovir (Chen *et al.*, 2020; Navé *et al.*, 1995; Navé *et al.*, 1992). Moreover, *coronaviruses* lack a viral kinase required for activation of these acyclonucleotide analogs (Chen *et al.*, 2020; Hible *et al.*, 2006). Despite these limitations, some market-available analogs, such as cidofovir, brincidofovir or favipiravir, do not require a viral kinase activation, and have shown *in vitro* activity against other RNA viruses (Ebola virus) and retroviruses (HIV-1) (Arias *et al.*, 2014; Dunning *et al.*, 2016; Garcia *et al.*, 1998; McCarthy *et al.*, 2016; Navé *et al.*, 1995). Thus, they may be drug candidates worth an exploration in the face of the findings in this study and the current SARS-CoV-2 outbreak.

## 4 Discussion

As proposed by Aller *et al.* (2018), computational approaches combining FBA and FVA to recover new metabolic antiviral targets are useful, especially in cases of new and emerging viruses, such as the SARS-CoV-2. In this study, we presented a host-virus integrated GEM using the human alveolar macrophage model *i*AB-AMØ-1410 as host cells and SARS-CoV-2 as virus. We identified potential targets for antiviral therapies using reaction knock-outs and host-derived enforcement approaches and analyzing their metabolic effects on host- and virus-optimized states by optimizing either for the host maintenance or the VBOF.

However, the VBOF constructed in this study only considers amino acids, nucleotides and energy requirements. It does not consider or include virus-host cell recognition, viral entry or the lipid envelope production or release (Timm and Yin, 2012). Especially the metabolic process of lipid envelope production of viruses can give further insight into potential targets for antiviral therapies. First studies with other *coronaviruses*, such as the human coronavirus 229E, suggest elevated and perturbed glycerophospholipids and fatty acids production rates in infected cells (Yan *et al.*, 2019). Yan *et al.* (2019) suggest the lipid metabolism regulation as a potential druggable target for coronavirus infections. Further information about the lipid metabolism of *coronaviruses* can enable the integration of lipids into the VBOF. Analyses with the adapted VBOF could highlight additional potential targets for antiviral therapies.

As their coding capacity is limited, *coronaviruses* strategically regulate host immune response, cell cycle, signaling and metabolism to create a favorable environment for viral replication (de Wilde *et al.*, 2015; Dirmeier *et al.*, 2020; Perlman and Netland, 2009). Accordingly, the viroids depend on cellular enzymes for the formation of progeny, which makes host cell resources a potential target to limit virion production (de Wilde *et al.*, 2015; Dirmeier *et al.*, 2020). The viral hijacking of the cellular metabolic pathways, such as glycolysis, nucleotide and lipid biosynthesis, may shift the environment of the virus to a proliferation promoting environment (Thaker *et al.*, 2019). In this study, we have also demonstrated that SARS-CoV-2 interferes with the host cell organisms, more precisely, the purine biosynthesis pathway to provide for the production of its biomass and, thus, replication.

In our study, we used a GEM. This type of model enables the analysis of metabolic changes under certain constraints. However, only the metabolic changes can be investigated. Further network reconstructions, such as dynamic signaling, regulatory or kinetic network models, can give further insight into changes in signal transduction, regulatory processes or kinetic properties (Bernhard, 2011) of virus infections. Ravindran *et al.* (2019), e.g. analyzed the effect of HIV-1 and hepatitis C virus infections using a large human signaling network. They demonstrated how the infecting virus could bring the dynamically organized host system into its control. Tan *et al.* developed a mathematical model describing the virus-induced interferon (IFN) signaling process. Dynamic analysis and numerical simulations led to the suggestion that a balance between viral replication and IFN-induced regulation is responsible for the dynamic behavior of virus-triggered signaling and also for antiviral responses (Tan *et al.*, 2012). Dynamic modeling of infections with *coronaviruses*, especially with SARS-CoV-2, could broaden the understanding of its effects on the host and give further insight into potential targets for antiviral therapies.

In this study, we used the already published *i*AB-AMØ-1410 GEM of human alveolar macrophages. This model does currently not include any genes or annotations. It is built upon the first human reconstruction Recon 1 (Duarte *et al.*, 2007). By now, the human reconstruction Recon3D is available with more than 10 000 reactions, 2000 genes and almost 6000 metabolites (Brunk *et al.*, 2018), including numerous annotations. Following the protocol of Bordbar *et al.* (2010), a new model of the human alveolar macrophage could be generated based on the newer version of the human reconstruction, Recon3D. The newly curated model could then be used to verify the findings from this study and to identify further targets.

As *coronaviruses* are reported to infect human alveolar macrophages, and a model for these cells was available, we integrated SARS-CoV-2 into this model. Nevertheless, SARS-CoV-2 is reported also to infect upper and lower respiratory tract cells, including pharyngeal regions (Chen *et al.*, 2020; Huang *et al.*, 2020). GEMs for bronchial epithelial cells and airway epithelial cells (AEC) are already available. Wang *et al.* reconstructed 126 human tissue-specific GEMs using the metabolic Context-specificity Assessed by Deterministic Reaction Evaluation algorithm. Those models are also built upon Recon 1. Furthermore, the models include fewer numbers of reactions (1242 and 1296, respectively) and lack a biomass maintenance function. Following the protocol for generati9.5 ng high-quality genome-scale metabolic reconstructions by Thiele and Palsson, meaningful models of human bronchial and AEC could be generated (Thiele and Palsson, 2010). However, generating a biomass maintenance function requires much data. Tools, such as BOFdat, can be beneficial for the generation of an appropriate BOF (Lachance *et al.*, 2019). These models can then be used to verify the potential antiviral targets that were found in alveolar macrophages.

The integrated host–virus model suggested the supplementation of L-isoleucine and L-lysine as a potential target for antiviral therapies, as well as the inhibition of the GK1. Since compounds that directly inhibit GK1 are already known, their evaluation and verification in cell culture experiments are required for fast responses to the current spread of SARS-CoV-2 worldwide.

## Funding

This work was supported by the Deutsche Forschungsgemeinschaft (German Research Foundation) under Germany’s Excellence Strategy—EXC 2124—390838134 and was supported by the German Center for Infection Research.

*Conflict of Interest*: none declared.

## Data availability

The computational model is accessible at https://identifiers.org/biomodels.db/MODEL2003020001.
